# Stem cells in ectodermal development

**DOI:** 10.1007/s00109-012-0908-x

**Published:** 2012-05-09

**Authors:** Salvador Aznar Benitah, Michaela Frye

**Affiliations:** 1 Centre for Genomic Regulation (CRG) and UPF, Dr. Aiguader 88, 08003 Barcelona, Spain; 2 Wellcome Trust Centre for Stem Cell Research, University of Cambridge, Tennis Court Road, Cambridge, CB2 1QR UK; 3 Institució Catalana de Recerca i Estudis Avançats (ICREA), Pg. Lluis Companys 23, 08010 Barcelona, Spain

**Keywords:** Organogenesis, Epidermal stem cells, Skin development

## Abstract

Tissue-specific stem cells sustain organs for a lifetime through self-renewal and generating differentiated progeny. Although tissue stem cells are established during organogenesis, the precise origin of most adult stem cells in the developing embryo is unclear. Mammalian skin is one of the best-studied epithelial systems containing stem cells to date, however the origin of most of the stem cell populations found in the adult epidermis is unknown. Here, we try to recapitulate the emergence and genesis of an ectodermal stem cell during development until the formation of an adult skin. We ask whether skin stem cells share key transcriptional regulators with their embryonic counterparts and discuss whether embryonic-like stem cells may persist through to adulthood in vivo.

## Introduction

Stem cells are defined by their ability to self-renew indefinitely but also to produce daughter cells that have different, more restricted properties. Pluripotent stem cells have the capacity to form all the body’s lineages, whereas multipotent stem cells generate all lineages that constitute an entire tissue or organ throughout adult life [1]. Whereas, these stem cell definitions are now widely accepted and stem cells have been described for a wide range of tissues, the origin of specific stem cell populations in the adult mammalian body is largely unknown [2]. Since they must arise in the embryo, it seems logical to assume that during development pluripotent stem cells give rise to more and more restricted cells, which then give rise to the different tissue-specific stem cells in a classic hierarchical model [2].

**Fig. 1:**
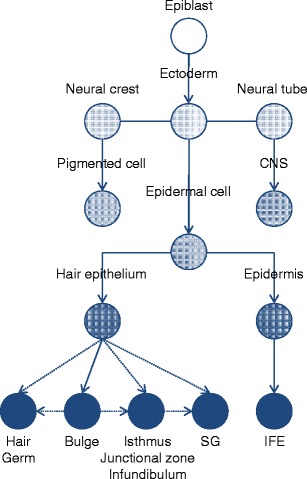
The developmental hierarchy for epidermal stem cell populations. The *dotted lines* indicate a putative relation between the populations. *CNS* central nervous system, *SG* sebaceous gland, *IFE* interfollicular epidermis

The first critical event for embryo patterning during mammalian development is the formation of a blastocyst. The blastocyst consists of three lineages, the trophoblast, hypoblast and epiblast. The epiblast generates the entire fetus and a single mouse epiblast cell, isolated at this stage and microinjected into another blastocyst, can contribute to all lineages [3]. Functionally, the preimplantation epiblast is the developmental ground state and is known to be the source of embryonic stem cells [4, 5]. However, the life of an epiblast is short during development. Epiblast cells lose their self-renewal capacity as soon as they turn into a cell of one of the three germ layers (Fig. 1); and also precursor cells of ectoderm, endoderm and mesoderm are not self-renewing indefinitely because they change identity during organogenesis. For instance, the origin of the hematopoietic stem cell in the mouse is well-known but cells capable of long-term repopulation of irradiated host animals are not present in the early embryo and only arise after about 10.5 days of development [6]. Thus rather paradoxically, indefinitely self-renewing stem cells do not exist in the early embryo up to the stage of organ formation [2, 7]. The question of whether pluripotent cells irreversibly turn into more restricted cells during development is debated. Through in vitro culture, it is possible to reprogram differentiated cells into an embryonic stem cell-like state and also the generation of pluripotent cells isolated from postnatal organisms has been reported [2, 8]. However, the existence of a comparable pluripotent cell in vivo is unlikely [9]. A model, which allows the frequent loss of stem cells through commitment to a more restricted fate during development, makes it very challenging to track the origin of one adult tissue stem cell down to the embryo. Under such a model, it is also unlikely that adult tissue stem cells and their embryonic original counterpart share many characteristic features, such as expression profiles; however they may share key transcriptional regulators. Below, we have made an attempt to follow a mouse embryonic cell through ectodermal development until formation of adult skin.

## First decisions of ectodermal cells and commitment to an epidermal fate

**Fig. 2:**
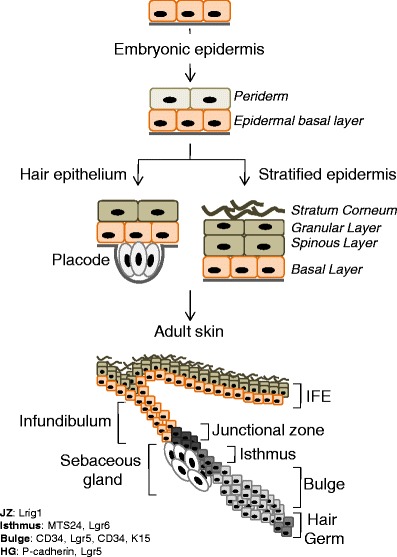
Epidermal structures formed during development until adulthood. The stratified epidermis is formed by E18.5 and gives rise to the interfollicular epidermis (IFE) and infundibulum in adult skin. The hair epithelium is initiated at around E14.5 by the placode or composed of bulge hair germ (HG), isthmus and junctional zone in adult skin. *Markers* for the respective epidermal compartments are indicated in the *left hand corner*

After gastrulation, the embryo surface consists of a single layer of neuroectoderm, which will form the nervous system and skin epithelium. Neural induction is positively enforced by extrinsic cues, including protein members of fibroblast growth factors (Fgf) acting in concert with inhibition of bone morphogenetic proteins (Bmp) [10]. In contrast, epidermal fate can be enforced by expression of bmp; and continual Wnt signaling blocks the response of epiblast cells to Fgf signals, permitting the expression and signaling of Bmp to direct an epidermal fate [11, 12]. The result of combinatorial Wnt, Fgf and Bmp signaling is a single layer of epidermal cells, covered by a transient protective layer called the periderm (Fig. 2). The function of the periderm is unclear but likely to form an early epidermal barrier to protect the developing skin from constant exposure to amniotic fluid. The periderm is shed once the stratification program is completed [13]. Since the periderm is a unique feature of developing epidermis, multipotent stem cells maintaining the periderm or periderm-promoting signals are lost over the course of stratification. In mice, ectodermal commitment to an epidermal fate is initiated at 8.5 days of development and the stratification program lasts about 10 days [14].

## Transcriptional regulators in the developing epidermis

Although dermal signals induce or repress a whole range of responsive genes in the developing epidermis, p63 is one of the earliest induced transcription factors associated with epidermal fate [14]. The p63 protein is a structural and functional homologue of the tumor suppressive transcription factor p53, and due to high sequence identity in their transactivation domains, p63 can transactivate p53-responsive genes [15]. Ablation of p63 during mouse development leads to the formation of truncated limbs and a block of ectodermal specification [16–18].

Although it can be argued that expression of p63 is not restricted to stem cells, it is an essential factor for the formation of an intermediate layer between the basal layer and the periderm, which is the earliest morphological sign of stratification [18–20]. The intermediate cell layer is later replaced by post-mitotic spinous layers [19]. In conclusion, p63 is a crucial factor allowing ectodermal stem cells to develop and survive. Similarly, another protein homologue p73, which is not expressed in epidermal cells, ensures the survival of neural stem and early progenitor cells during development [21, 22].

The p63 gene encodes several protein isoforms generated by alternative splicing and how or whether specific isoforms control epidermal stem cell fate remains unclear [23]. The most abundant isoform in the epidermis (ΔNp63α) lacks a transactivation domain, and accordingly fails to induce apoptosis and inhibits p53 transcriptional activity [24]. The full-length TAp63 isoforms are the first to be expressed during embryogenesis and are required for initiation of epithelial stratification but TAp63 isoforms must be counterbalanced by ΔNp63 isoforms to allow cells to respond to signals required for maturation of embryonic epidermis [18]. In zebrafish, ΔNp63 over-expression blocks neural development and promotes non-neural development [25]. Thus, the lack of ectodermal specification in p63 null mice might be due to a combination of a failure to establish and maintain epidermal stem and progenitor cells.

Although the precise function of the different p63 isoforms in stem and progenitor cells is debated, p63 clearly plays a major role in embryonic development of ectodermal lineages [23]. Heterozygous mutations in the human p63 gene are responsible for several ectodermal dysplasia syndromes, which are congenital disorders characterized by abnormalities of two or more ectodermal structure, including hair, teeth, nails and sweat glands among others [23, 26].

Another transcription factor required to maintain an undifferentiated and proliferative state of epidermal progenitors in both the developing and adult skin is the Yes-associated protein (YAP1). YAP1 is a proto-oncogene from the Hippo pathway; nuclear YAP1 specifically marks progenitor cells in the developing epidermis and its deletion results in epidermal embryonic hypoplasia [27, 28].

## Epigenetic regulators in the developing epidermis

Robust changes in gene expression during development and cellular differentiation are often achieved by a tight interplay between transcription factors and chromatin modifying enzymes [29, 30]. Setd8, the sole enzyme to catalyze the formation of mono-methylated histone 4 at lysine 20, is essential for survival of basal ectodermal cells prior to stratification and its deletion results in a phenotype similar to that observed upon deletion of p63 [31]. Due to redundancies, the direct roles of other chromatin modifying enzymes in regulating epidermal progenitors are often complex and difficult to define.

Ezh1 and Ezh2 mediate tri-methylation of histone 3 at lysine 27 (H3K27me3) and are part of Polycomb Repressive Complex-2 (PRC2). Deposition of H3K27me3 in promoter regions correlates with transcriptional repression and is essential for lineage commitment of embryonic stem cells into the three germ layers [32, 33]. In the epidermis, only complete loss of the H3K27me3 by deletion of both Ezh2 and Ezh1 causes de-repression of non-epidermal lineage markers [34, 35]. Intriguingly, the PRC2 complex is also required to balance epidermal stem cell proliferation in adult skin, as shown by deletion of Ezh1/2 and Jarid2 [34, 36].

## Regulators of the epidermal stratification process

One of the first steps to complete the stratification program during development might be the direct transcriptional activation of the genome organizer Satb1 by p63 [37]. Through remodeling of chromatin, Satb1 causes transcriptional activation of genes located to the epidermal differentiation complex, a gene locus essential for skin maturation [37, 38]. During the differentiation program, epidermal progenitors then detach from the basal membrane and switch to a post-mitotic suprabasal state. This switch is achieved through asymmetric as opposed to symmetric cell divisions; and further involves Notch signaling pathways, polarized cytoskeletal and adhesive changes as well as expression of proteins that will ultimately form the impermeable epidermal barrier; processes that have been extensively reviewed elsewhere [14, 39–44].

Transcriptional regulators inducing the basal to suprabasal epidermal switch during embryogenesis involve the retinoblastoma family members p107 and p130, both of which are required for establishing a quiescent cell cycle state of suprabasal cells [45]. The CCAAT/enhancer binding proteins C/EBPα and C/EBPβ are expressed in the first suprabasal layer, where they stimulate the onset of differentiation by repressing p63 [46]. Post-transcriptonal repression of p63 during stratification is achieved by the microRNA miR-203 [47].

During stratification the epidermal cells progressively differentiate as they move upwards and form the spinous, granular and finally the dead, impermeable cornified layer of the skin (Fig. 2). After completion of the stratification program, cells located in the basal layer of the interfollicular epidermis maintain its structure throughout adult life; and under homeostatic conditions, they can do so independent from any other stem cell population found in skin to date [48–50].

## The development of a hair follicle

About half way through the stratification program at embryonic day 14.5 ectodermal stem cells can adopt an alternative fate to epidermis, the formation of a hair follicle (Figs. 1 and 2). Commitment to a follicular epithelium starts with the formation of a placode (Fig. 2). Placode formation is dictated by signals sent from the underlying mesenchyme, the dermal cells. The mesenchymal cells aggregate immediately underneath the epidermis and mark the location of the new hair follicle [51]. These aggregates or dermal condensates are the precursors of the dermal papilla, the permanent mesenchymal part of a hair follicle [52]. The occurrence of dermal aggregates, and thus the formation of hair follicles, is controlled in a strict spatiotemporal manner and the signals involved in this process have been extensively reviewed [51–55].

One of the first essential events to form a placode is the activation of Wnt signaling in the epidermis; expression of the Wnt inhibitor Dickkopf-1 abolishes the development of hair follicles [56–58]. Activation of Wnt signaling in the hair follicle epithelium is followed by expression of Sonic hedgehog (Shh), which is important for the early development and maturation of the dermal papilla [59, 60]. Key dermal factors also include Fgf and subsequent inhibition of Bmp [61, 62]. Interestingly, Fgf signaling collaborating with Bmp inhibition induced neural induction earlier in development, indicating that the secretion of a highly specialized cocktail of factors from the underlying mesenchyme is temporally and spatially controlled. Accordingly, the dermis not only dictates the formation of appendages but also timing, spacing, size and type [63].

Maturation of the placode is co-ordinated by Wnt targets such as the Leucine-rich repeat containing G-protein coupled receptor 4 (Lgr4) and EDAR, both of which are required for the proper initiation and maintenance of the primary hair follicle placodes [64, 65]. Once organized, the placodes start proliferating and grow downwards, a process that is dependent on expression of Shh in the proliferating epithelial cells at the distal tip of the developing hair follicle [59, 66, 67]. Notably, the role of Shh in hair follicle development is epidermal cell autonomous because hair follicle formation is initiated and the dermal condensate is formed in mice lacking Shh [68]. The down-growing hair follicles generate the first hair germs at embryonic day 15.5, which will develop into the epithelial part of the hair follicle and then elongates into hair pegs at embryonic day 16.5 to 17.5.

The correct directional down-growth of the placode is determined by specific sets of microRNAs; conditional deletion of the miRNA processing enzymes Dicer or Dgcr8 does not impair placode formation, but causes placode evagination into the embryonic epidermis [69, 70]. Interestingly, placode evagination is also observed in response to impaired integrin and YAP1 signaling pathways, indicating that polarity of embryonic hair growth is regulated through both transcription and cell-matrix interactions [27, 71]. During down-growth, the leading front of the hair follicle (matrix) remains proliferative through its interaction with the dermal papilla, whereas reduced adhesive properties and proper polarization of dermal–epidermal interactions allow migration into the dermis [45, 72, 73].

At embryonic day 18.5, the inner root sheath develops into the hair channel and the outer root sheath maintains contact with the basement membrane [52]. At birth, the most mature hairs begin to break the surface and maturation continues through to the first postnatal week [55]. At postnatal day 17, hair morphogenesis ends and the first adult hair cycle begins. In adult skin, the hair follicles keep undergoing cyclic bouts of growth (anagen), apoptosis-mediated regression (catagen) and rest (telogen) [74, 75].

## Origin of hair follicle stem cells

The origin of an adult hair follicle stem cell is particularly difficult to define. The classical view of a hair follicle stem cell is a slow cycling cell, which exhibits long-term contribution to all hair compartments. Such multipotent hair follicle stem cells are located in a special microenvironment called the bulge (Fig. 2) [40, 76]. The establishment of the quiescent bulge takes place early during postnatal hair follicle morphogenesis, and depends on signals that are already present in the embryonic placode [77]. However, recent studies on the hair follicle have uncovered diverse and cycling populations of stem cells outside the bulge region (isthmus and junctional zone; Fig. 2), which can also act as multipotent stem cells in stress situation, such as injury [48]. These studies established the concept of the existence of several classes of epithelial stem cells in the hair follicle [78, 79]. How these stem cell populations relate to each other is unclear but recent evidence suggests a hierarchical organisation with a quiescent bulge stem cell at the base [80].

One strategy to identify a potential common founder population of adult hair follicle stem cells is the genetic marking of cells, which allows the tracing of all daughter populations. Labelling of Shh-expressing cells in the placode showed that the progenies can indeed generate all structures of a hair follicle [50], indicating that the Shh-positive placode cell is the origin of all hair follicle stem cells. In contrast, progeny of stem cell populations in the adult hair follicle contribute to more restricted lineages of hair follicle during homeostasis [48]. One Shh-dependent transcription factor expressed in the placode is Sox9. Progeny of Sox9-expressing cells also contribute to all hair lineages and ablation of Sox9 leads to a failure to generate hair and sebaceous glands and the bulge stem cell niche is never formed [77, 81]. Whether a placode-like cell persists throughout adult life and how they may relate to cycling stem cell populations in the hair follicle remains unclear.

Molecular regulators that control both embryonic and adult hair follicles are rare but expression profiling of placode cells compared to other epidermal populations revealed a couple of additional transcriptional regulators enriched in the placode, which are associated with postnatal genetic hair defects: Cutl1, Gli1, Hoxc13, Lhx2 and Runx1 [53, 55, 82–84].

## The development of a sebaceous gland

Together with the hair, the majority of sebaceous glands are an integral part of a pilosebaceous unit; although some glands can be found without an associated hair follicle [85]. Sebaceous glands are functionally important to maintain hair; and lack of sebaceous glands can be associated with scarring alopecia [86, 87]. Sebaceous glands form late in mouse development and appear when follicles elongated into hair pegs. Although absence of Sox9 inhibits the formation of an early bulge and sebaceous glands, the first sebaceous cells in the developing hair follicle are not Sox9 positive but arise from Lrig1-positive cells in the hair follicle [77, 88]. During homeostasis in adult mouse skin, Lrig1-positive cells contribute to the infundibulum and the sebaceous glands [89].

## Concepts of homeostatic stem cell self-renewal

Tissue homeostasis is a balance between stem cell self-renewal and the generation of committed daughter cells. As in many other tissues, a single stem cell that is the origin of all epidermal lineages in a non-perturbed condition has still to be identified in adult skin. Whether the absence of a single stem cell at the base of a potential hierarchy is due to technical limitations in the respective studies or simply reality is debated. However, the notion that tissue homeostasis is not achieved by a single stem cell but at the level of stem cell populations is rising. Under this model, stem cell fate within a population is stochastically determined, meaning that the fate of an individual stem cell is random, whereas the dynamics of a population unfolds in a predictable manner [90]. Importantly, this model allows the loss of individual stem cells and a ‘shift’ of properties within stem cell populations determined by intrinsic and extrinsic cues. Such a model is attractive to explain stem cell behavior during development and has been suggested for a number of adult tissues, including the intestine, epidermis and blood [90, 91]. For instance, genetic marking of single cells in the mouse epidermis revealed that a stochastic cell population with random fate to produce stem or differentiated daughter cells is sufficient to maintain the epidermis in the long term [90, 92, 93]. As mentioned above the identity of the cell at the very base of this hierarchy is debated and its absence might be due to technical limitations of the assay [94].

In the hair follicle, self-renewal and differentiation processes might be differently regulated. During development, hair follicle cells self-organize to anatomical patterns by coordinating few morphogenetic signals [95–97]. Activation of Wnt in the dermal papilla is one of the strongest inducers of anagen and activators of hair follicle stem cells to enter proliferation and differentiation pathways [98]. Interestingly, spontaneous activation of Wnt in individual dermal papillae, however, does not translate into anagen entry as long as the number of Wnt activated dermal papillae in neighboring hair follicles is below five [96]. Within the hair follicle bulge, stem cells divide infrequently and enter quiescence in telogen, when single bulge cells migrate out of their niche to undergo proliferation as progenitors before they differentiate into hair [79, 99].

## Summary

Although some progress has been made to identify the origin of tissue specific stem cells, there is no clear evidence that an embryonic-like stem cell is maintained in adult skin. It is however clear that both quiescent and cycling stem cell populations found in skin have restricted differentiation potential under homeostatic conditions but can give rise to all epidermal lineages after injury or insult [40, 48]. How this increase in plasticity is achieved remains to be investigated but might either involve activation of a very rare single stem cell or de-differentiation processes of progenitors.
